# Family Experiences of Loss and Bereavement in Palliative Care Units during the COVID-19 Pandemic: An Interpretative Phenomenological Study

**DOI:** 10.3390/healthcare12171763

**Published:** 2024-09-04

**Authors:** Maria João Mateus, Luís Simões, Amira Mohammed Ali, Carlos Laranjeira

**Affiliations:** 1 School of Health Sciences, Polytechnic University of Leiria, Campus 2, Morro do Lena, Alto do Vieiro, Apartado 4137, 2411-901 Leiria, Portugal; 12817@ulsra.min-saude.pt; 2 Palliative Care Inpatient Unit, Local Health Unit of the Aveiro Region, Visconde Salreu Hospital, Rua da Agra 23, 3865-206 Salreu, Portugal; 3 Department of Psychology, Local Health Unit of Coimbra, Praceta Professor Mota Pinto, 3004-561 Coimbra, Portugal; 18508@ulscoimbra.min-saude.pt; 4 Department of Psychiatric Nursing and Mental Health, Faculty of Nursing, Alexandria University, Smouha, Alexandria 21527, Egypt; amira.mohali@alexu.edu.eg; 5 Centre for Innovative Care and Health Technology (ciTechCare), Polytechnic University of Leiria, Campus 5, Rua das Olhalvas, 2414-016 Leiria, Portugal; 6 Comprehensive Health Research Centre (CHRC), University of Évora, 7000-801 Évora, Portugal

**Keywords:** bereaved relatives, palliative care, death, qualitative study, COVID-19, Portugal

## Abstract

The COVID-19 pandemic significantly interrupted the grieving experiences of bereaved families and drastically changed their ways of dealing with loss. Our study aims to gain an in-depth understanding of the experience of bereaved relatives of patients who died in palliative care units during the COVID-19 pandemic. The phenomenological research design included sixteen family members of hospitalized palliative patients who died from November 2021 to June 2022. The study involved conducting qualitative in-depth semi-structured interviews with family members 12–24 months after the death of their loved ones. The interviews aimed to gather information about the experiences of the families both before and after the death. The COREQ guidelines were applied in the study. Participants were mainly female (n = 13) with a mean age of 47.25 (SD = 12.58). Data were analysed using the Interpretative Phenomenology Analysis (IPA). The following three categories were identified: (1) navigating loved ones’ final weeks and days (troubled deaths); (2) the last farewell was robbed; (3) looking for adjustment after loss. One overall main theme emerged, which was as follows: “Struggling between stolen moments and painful losses to get back into the flow of life”. This study provides novel insights into end-of-life care and bereavement from the perspectives of family. Our findings suggest that developing and promoting family-centred culture can lead to compassionate palliative care focused on a myriad ways of affirming that their loved one matters.

## 1. Introduction

The worldwide COVID-19 pandemic has been characterized as a hostile time for death, with concerns voiced about a significant deviation from a palliative care strategy [1]. Visiting limitations, while essential for safeguarding vulnerable patients receiving palliative care from potential virus transmission, have been recognized as a significant contributor to the rise in people experiencing solitary deaths [2,3]. There have also been concerns expressed over the accessibility of specialized palliative care services throughout the pandemic period. In several countries, palliative care units (PCUs) enforced complete prohibition of visits, while others were more flexible. The absence of effective rules for family visitation, especially during the final stages of life, led to the widespread acceptance of utilitarian and paternalistic approaches that disregarded the needs of patients and their families in the provision of palliative and end-of-life (EoL) care [4,5]. Scarce research has been conducted on the impact of restricted visitation on families who have lost a loved one in EoL care.

A systematic analysis of global evidence indicates that efforts to decrease the risk of infection have led to a decrease in the participation of community nursing and hospice services [6]. Furthermore, it has been discovered that patients and their families perceive healthcare systems as lacking resources and as potential sources of viral transmission. Consequently, patients/family members decreased their interactions with healthcare services and reported negative experiences communicating with healthcare professionals (HCPs) during this time [7,8,9]. Visiting limitations have been proven to increase wasted opportunities for quality time and farewells with dying family members. Dissatisfaction due to inadequate communication by healthcare workers caused significant distress to family members [10,11]. Previous research suggests that the heightened demands placed on families to provide care had detrimental effects on their well-being, intensifying levels of anxiety, sadness, weariness, sleep disruption, and feelings of social isolation and loneliness among family caregivers [12,13,14]. The pandemic also adversely affected the financial well-being of family caregivers [14]. Additional research indicates that patients and their families were not adequately provided with spiritual or emotional assistance throughout the last stages of their lives [2].

Two years after the pandemic, van Schaik [15] revealed that the COVID-19 pandemic significantly transformed how bereaved family members undergo and articulate their grief, encompassing both adverse and beneficial consequences. Deaths during the COVID-19 pandemic were marked by poor bereavement outcomes, with significant repercussions for mental and spiritual well-being [15]. Nevertheless, family members have also shown indications of resilience in dealing with their grief and ascribing significance to the death of a cherished someone under challenging situations [15].

Research on bereavement during the pandemic identified the following two significant aspects that can alleviate suffering and help families with coping and adjusting to the loss of a loved one: (a) effective communication provided by HCPs about a loved one’s illness; and (b) active participation of family members in the decision-making process, which includes enabling them to communicate with the patient [16,17,18]. Nevertheless, due to the substantial workload during the COVID-19 pandemic, HCPs were occasionally unable to fulfil these requirements. A significant number of HCPs were compelled to operate in inadequately equipped facilities, resulting in their frequent inability to fulfil patients’ needs or communicate with patients’ relatives to provide updates on the health status of their suffering family members [19]. The pandemic presented unforeseen and unparalleled obstacles in society, and HCPs encountered moral and ethical quandaries during this arduous period [5,20]. Conversations about EoL matters were hindered, and there was an increase in the number of individuals dying without any family members present. This was mainly due to restrictions on visitations and travel, which affected families that live far apart [21].

Enduring the loss of a cherished individual is one of the most arduous challenges in life. Hence, the lives of bereaved families and individuals are completely disrupted. The theory of meaning reconstruction by Neimeyer [22] argues that grieving is a cognitive process of meaning-making following a loss, and that the loss itself disrupts the consistency of an individual’s self-narrative. The process of grief is intricate and subjective, exhibiting variation among individuals and within the same person at different times. This process encompasses psychological, physiological, and social responses, including a variety of emotions such as hopelessness, sadness, culpability, loneliness, and exhaustion [23]. A variety of factors, including the personality and life story of the bereaved, their relationship to the deceased, the circumstances of death, the support provided to the bereaved, and cultural practices associated with death and grief, can influence these typical responses to grief [24].

Scholars have examined the consequences that occur after the loss of a loved one, expressing fears that the pandemic led to an increase in complex and prolonged grief [25]. Evidence indicates that complex grieving is linked to unfavourable opinions regarding the quality of the dying process and a lack of readiness for death [26,27]. Complex grief is a matter of public health that is associated with various negative health consequences, including major depressive disorder, post-traumatic stress disorder, substance abuse disorder, and other detrimental mental health outcomes, such as the risk of disenfranchised grief, prolonged grief disorder, suicidality, decreased quality of life, and overall functional impairment [6,28,29]. Indeed, studies indicate that individuals who experienced the loss of a loved one during the pandemic had more intense grieving reactions compared to those who lost someone due to natural causes [30]. Indeed, the grieving process is hindered by simultaneous limitations on post-death rituals and funeral assemblies [28]. Insufficient access to and limited availability of specialized bereavement care have been recognized as obstacles in some countries [31].

Although attempts have been made to incorporate the use of phone and videoconferencing to facilitate communication [32,33], this cannot sufficiently replace the physical presence of family members, especially for palliative patients [34]. The pandemic posed significant problems to various aspects of modern healthcare, including the provision of palliative care [32]. Although we aim for person-centred models of care that prioritize the needs of patients, during times of uncertainty, the underlying focus on hospital centrism becomes a reality [35].

Although COVID-19 is no longer officially categorized as a pandemic, it has exerted a substantial influence for an extended period. Indeed, there is a scarcity of information addressing the impact of the imposed limits on families who experienced the loss of a loved one due to COVID-19. Furthermore, the impact on relatives of the overwhelming number of patients requiring hospital care during this pandemic, coupled with a high fatality rate, remains mostly uncertain. Hence, investigating the firsthand accounts of family members who lost a loved one during the pandemic can yield valuable insights into the strategies and adjustments used by families in coping with the death of a family member during the COVID-19 pandemic. Moreover, healthcare professionals and policymakers must comprehend the requirements of families during a pandemic in order to formulate effective ways to address these needs in the future and make recommendations for EoL care and bereavement support.

Most of the available studies come from Western Europe [18,36,37,38], the United States of America [39,40], New Zealand [41,42], Australia [43] and Canada [44] and, consequently, it seems important to highlight how the cultural context can determine different perspectives on the phenomenon. To date, there are no known studies in Portugal exploring how bereaved family members experienced EoL support and its implications for the bereavement process. In Portugal, as in other southern European countries, mourning practices are a significant component of Christian burial rites, typically involving a sizable gathering of family and friends. Even though these rituals necessitate a significant amount of physical interaction with the deceased and with one another, the pandemic has either reduced their duration or prohibited them entirely [45].

Given the lack of comprehensive qualitative evidence regarding the diverse facets of pandemic bereavement experiences, our research question was “What is the meaning of the lived experience of loss and bereavement presented by family members of patients who died in palliative care units during the COVID-19 pandemic in Portugal?”. Therefore, our aim is to gain an in-depth understanding of the experiences of bereaved relatives of patients who died in palliative care units during the COVID-19 pandemic.

## 2. Materials and Methods

### 2.1. Study Design

This is an inductive qualitative study anchored in interpretative phenomenological analysis (IPA) aimed at interpreting the meanings of human phenomena and the perceptions that people have of what they experience. This idiographic approach is concerned with a holistic exploration of lived experience, and how people make sense of that experience [46]. It also recognizes a subject’s intersubjectivity from an ontological stance, using the hermeneutic circle to understand lived experience [47]. Philosophically, the research is underpinned by critical realism [48], which is particularly valuable for comprehending the mechanisms behind events and examining the impact of surrounding circumstances on outcomes in a real-life context. This study focused on individuals’ decision-making processes during times of crisis, specifically in the context of EoL situations and a worldwide pandemic. We also examined how these individuals responded to changes in the care and support systems they relied on.

The study follows the consolidated standards for reporting qualitative research (COREQ) recommendations [49] (Supplementary Table S1).

### 2.2. Sample and Recruitment

The study involved bereaved family members who lost their loved ones, between November 2021 and June 2022, in two specialized palliative care inpatient units integrated into public hospitals in the central region of Portugal. During this period, restrictive measures were in force to mitigate the spread of COVID-19, particularly restrictions on the number and duration of visits, the use of Personal Protective Equipment (PPE), and the mandatory negative test for SARS-CoV-2.

The inclusion criteria were as follows: (1) bereaved family members of palliative patients hospitalized during the restriction measures associated with the COVID-19 pandemic; (2) aged ≥ 18 years; and (3) understanding and speaking Portuguese. Bereaved family members were excluded if they revealed moderate to severe cognitive deficits as screened using the Short Portable Mental Status Questionnaire (SPMSQ; score of ≥5 or a score of ≥6 or higher for those with only a grade school education) [50].

With the intention of promoting maximum sample variation, we decided not to impose any restrictions regarding the type of bond between the bereaved family member and the deceased person. The essential criterion was being a significant person to the participant, regardless of the degree of kinship and whether or not there was a formal family bond.

The sample size was established when data saturation occurred [51]. Data saturation was deemed to have been attained when the interviews with the participants no longer yielded any novel themes.

### 2.3. Data Collection

Participants were initially recruited through a purposive sampling technique via the project’s Facebook page and the author’s contact network or recruited later by chain-referral (snowball) sampling. Invitations to participate were sent six months after the loved one’s death to honour the anticipated grieving process. Participants could then directly contact the lead researcher (M.J.M.) if they wished to participate in an interview. Subsequently, a brief description of the study was presented (i.e., objectives, procedures, estimated time of interview and general characteristics of participation). Then, the potential participants received a letter introducing the study. After collecting the consent form for participation in the study, the interview was scheduled.

Data were collected in-person, between June 2023 and November 2023, in a location according to each participant’s preference. Most of interviews took place in a private room at the local PCU or at the participant’s home. They had the option of having a support person, but none chose to do so. All interviews were conducted in European Portuguese by a trained interviewer (M.J.M.).

Data from the participants were collected through in-depth semi-structured interviews performed in the Portuguese language. The experiences of bereaved relatives who lost a loved one to COVID-19 were gathered to understand their lifeworld and lived experiences of the phenomena. The interview guide was developed based on the study objectives and supported by previous literature [16,18,36] and included two parts. The first part gathered sociodemographic data and characterization of the context that preceded the bereavement, including gender, age, marital status, education, professional occupation, type of relationship with the deceased, family support, time after the loss and previous losses of significant people. The second part included questions dealing with the following main topics: (a) experience of disease progression in the PCU; (b) communication flow with the patient, family and care team; (c) experiences of loss and bereavement; and (d) emotional responses to restricted rituals and the impact on individuals’ lives and their strategies for managing the situation. A pilot interview was conducted by an interprofessional research team to evaluate the interview guide, and no changes were made; thus, the pilot interview was included in the study.

After the initial presentations, the participant was invited to share their narrative, using the following question: “Tell me about your experience during the period in which your family member was admitted to the PCU?”. Follow-up questions were used as prompts during the interview to explore the family members’ experience of end-of-life care and the bereavement process, including the following: “What was important? What helped? What was difficult?”.

Field notes were taken (aspects such as tone of voice, gestures and body posture, among others) after each interview, which allowed us to obtain a broader and more precise understanding of the phenomenon under study [52]. Each participant was interviewed once. The interviews lasted an average of 72 min (ranging from 30 to 145 min) and were digitally recorded and transcribed in full by the first author (M.J.M.). The data collection and analysis process occurred concomitantly. After thirteen interviews, the first and last author agreed that data saturation was reached, as no new perspectives on the main themes in the interview guide were presented in the interviews. Nonetheless, three more interviews were made to ensure data saturation.

### 2.4. Data Analysis

The data analysis follows the core tenets of interpretive phenomenological analysis (IPA), which is characterized by its adaptable and iterative nature, as opposed to a rigid and linear approach [47]. IPA involves the following sequential steps: (1) reading and re-reading each transcript, (2) making initial notes that represent significant aspects of the analysis of each fragment, (3) identifying emerging patterns based on initial notes, (4) identifying connections between these emerging patterns, (5) moving on to the next case, (6) repeating steps 1 to 4 for each case, and (7) identifying commonalities across all cases [53].

In line with the “double hermeneutic” of the theoretical foundations of the IPA, we analysed how each participant conveyed and understood the lived experience of bereaved families within the context of the COVID-19 pandemic. Consistent with this idiographic approach, we thoroughly examined each case on an individual basis prior to assessing the similarities and differences among cases. The hermeneutic circle facilitates non-linear analysis, allowing for the continuous incorporation of new ideas into our thinking. The determination of when the interpretation was deemed satisfactory was left to the researchers [54].

To manage and store all the data, the qualitative data analysis software WebQDA (Version 3.0, University of Aveiro, Aveiro, Portugal) was used. The first and last author coded all interviews, and discussions among researchers produced a consensus. The essential structure of the lived experience was organized into themes and supported by fragments obtained in the interviews. Only after the analysis process were the textual quotes translated into English to maintain the original meaning.

### 2.5. Trustworthiness and Reflexivity

We followed Tracy’s eight criteria for quality in qualitative research [55], which including the following: “(a) worthy topic, (b) rich rigor, (c) sincerity, (d) credibility, (e) resonance, (f) significant contribution, (g) ethics, and (h) meaningful coherence” (p. 839). Worthy topics frequently arise from discipline interests and should be theoretically or conceptually compelling. In fact, the current qualitative study is relevant, timely and significant to understand the silent voices of bereaved families. The assessment of rigor is based on adherence to established procedures for data gathering and analysis as well the choice of a maximum variation sample. Sincerity was achieved because the researchers engaged in self-reflexivity prior to entering the field by engaging in introspection, evaluating their own biases and motivations. A detailed description was crucial for establishing credibility in the qualitative research. It involved providing detailed explanations that uncover culturally specific meanings and provide specific details. Likewise, we used researcher triangulation by seeking input during data analysis and producing a research report (member reflections). To achieve resonance, researchers engaged in practices that promoted empathy, identification, and reverberation of the research by readers with no direct experience with the topic discussed [55]. In this sense, an aesthetic and schematic presentation of lived experience was made. This strategy allowed the study’s potential to be valuable and transferable across similar other contexts or situations. Exploring the practical, educational and methodological implications contributed to the study’s resonance. All procedural and relational ethics procedures were followed in order to recognize and value mutual respect, dignity, and connectedness between the researchers and participants. Finally, meaningful coherence was achieved through an interconnection between the literature reviewed, research foci, methods, and findings.

In line with qualitative research, the reflexivity of the research team was also a valuable tool in enhancing rigor and clarifying the lenses used to arrive at certain interpretations [56]. The first author (M.J.M.), a female nurse with seven years of professional experience in palliative care, was even involved in monitoring EoL patients during the COVID-19 pandemic. She also experienced family member loss during the COVID-19 pandemic, which made her particularly empathetic to the participants’ experiences. The second author (L.S.) has a background as a clinical psychologist with experience in palliative care. The third author is a scholar and a specialist in mental health nursing (A.M.A.). Finally, the fourth author (C.L.) was the project’s scientific supervisor, with clinical and pedagogical experience in palliative care, and is an expert in qualitative research.

### 2.6. Ethics

The study was conducted under the principles of the Declaration of Helsinki and after approval by the Local Ethics Committee (52/01/2023/CES). Before each interview, free and informed consent was obtained in writing and the identity of each participant was preserved by assigning an alphanumeric code. Participants were informed that they had the option to withdraw from the study at any point. Additionally, they were advised that other than sharing their experience, there were no additional advantages of participating. No monetary reward was provided. All audio files, field notes, and transcriptions were transferred to a secure, password-protected drive accessible only by the first author (M.J.M.). After completion of the study, these data will be preserved for a period of one year, and then destroyed.

Given the potentially emotional topic, participants were informed that they could pause or end the interview at any time, without having to provide any justification. Strategies were also implemented to manage any participant distress by providing immediate support by the interviewer and referring participants to specialized grief support. Apart from occasional pauses in the interview flow, there was no need to implement the planned support strategies.

## 3. Results

### 3.1. Sample Characteristics

Sixteen bereaved family members participated, mostly female (n = 13), aged between 22 and 72 years old (M = 47.25; SD = 12.58). The majority completed higher education (n = 6). Regarding the type of relationship with the deceased, the majority were children (n = 8). The time elapsed from the loss to the interview ranged from 12 to 24 months. Only four participants had no history of significant previous losses. Sample description is depicted in Table 1.

### 3.2. Overview of Findings

**Figure 1:**
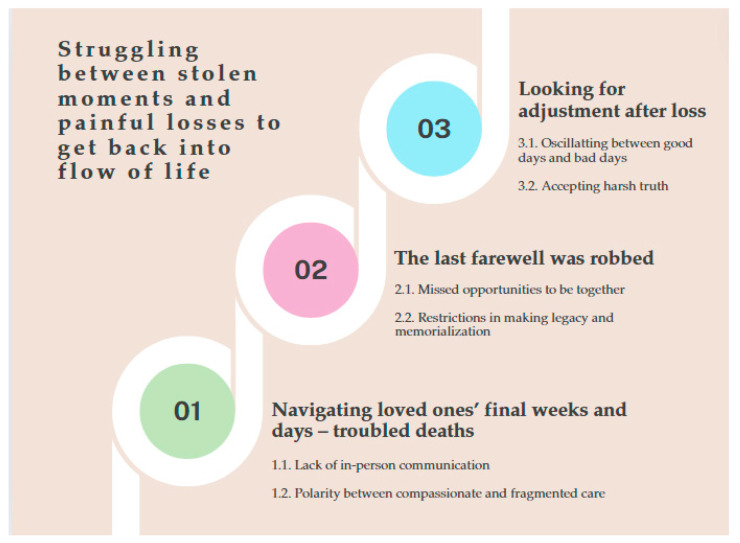
Overview of the essential meaning of the participants’ experience.

The analysis of interview data provided valuable understanding on the experiences of families who lost a loved one in palliative care settings. The essential structure of the phenomenon was identified as “Struggling between stolen moments and painful losses to get back into flow of life”. The essence of the phenomenon is presented as three theme clusters that highlight the experiences of the families both before and after death. The essential meaning of the participants’ experience is shown in Figure 1.

#### 3.2.1. Navigating Loved Ones’ Final Weeks and Days (Troubled Deaths)

Bereaved relatives experienced an emotional roller coaster while their loved ones were unwell. Although participants placed their trust in the staff, they experienced an inconsistent daily routine and were constantly waiting for updates regarding their loved one’s condition at the hospital. They experienced a sense of powerlessness when they lacked control over events, leading to sentiments of fear, solitude, and vulnerability.

(a) Lack of in-person communication

The last moments of an individual’s existence and their death, whether it be a family member or a close friend, is a challenging period. Individuals frequently report communication challenges with healthcare professionals, such as obstacles when attempting to reach staff and obtain accurate information regarding their loved one’s condition. In this sense, some relatives had feelings of frustration and anger due to the lack of crucial information regarding their terminally ill family member.

P3: *Information was scarce because there was no face-to-face contact with the team. I was very angry*… *I was only contacted to announce the death.* P3 also states that it would* have been important for at least one person, a family member, to have been able to make the daily visit. There are families who don’t even care*… *going or not going, for them it doesn’t matter, for us who have always been very close it would make all the difference.*

P7: *We were never told that this or that would happen. We were never told ‘prepare for this, prepare for that’*.

P8: *I felt a little upset with the team because I was there, and no one told me what was happening*… *No one told me that my grandfather was in the last hours or days of his life*.

Hoping to understand the ill family member’s evolution, P16 mentions that often there were long waiting times to contact the team and that he sometimes felt it was an overload, as shown in the following statement: *The waiting time made me anxious… the professionals answered my questions, but I felt there was no availability. Because my nerves were all on edge. But sometimes I noticed… and then they made me feel like ‘am I really that annoying’? Okay, I’m boring, but I need to know.* P16 also added that although communication was not ideal, she felt supported: *I was heard… But that’s the question, I understand that the time was difficult for everyone and involvement, the gestures, the tone of the voice was not always as sweet as a person perhaps needed at the time.* A similar experience was reported by P11: *we were always well informed about what was being done, what was to be done. This was very well explained and, therefore, very well accepted… because the team was open to explaining everything… However, it was more difficult to obtain information in person than over the phone.*

On the other hand, bereaved relatives often perceived staff members as lacking sensitivity to include families in EoL decisions. Participants reported not having participated in advance care plans, although P2 highly valued the relationship of total trust with the team: *We never talked about end-of-life decisions, but we always had the perception that the medical team that accompanied us, the entire team, would always be doing what was best and we were at ease and trusted them 100%. Because we trusted the team 100%.* In the case of P16, a feeling of abandonment emerged: *Maybe, if they had told me: ‘let’s gradually reduce the measures…’ they just told me that they had suspended therapeutic measures… It’s complicated… I felt like they were giving up…*

(b) Polarity between compassionate and fragmented care

Participants judged the healthcare system to be in a state of emergency, as the staff occasionally lacked the capacity to respond to inquiries and offer information. Some participants who were granted permission to see their ill loved ones in the PCU also had their social interactions restricted to comply with communal health requirements. At this point, P6 stated: *There were restrictions (facemasks, gloves, gowns), which had to be complied with because we were during the pandemic. But I always had as much time as I wanted. I went in the morning, and then I went in the afternoon (…) but of course I didn’t abuse it either. Within the rules I knew there was some flexibility. However, other family members were unable to be present.*

Some bereaved relatives had the expectation that the PCU would ensure safety and compassionate care of their beloved family members. Although participants relied on the medical system, they felt a sense of powerlessness.

P7: *What was most important for us was knowing that there were many rules to follow… we always, always, always felt enormous support from the team for everything*.

P6: *I distinguish Palliative Care from other medical services, because I saw care for the patient and care for the family. It’s not just the patient who is important, the family is important too… I trust them, but no one can replace the role of the family in those moments of affection that fill the souls of those who are suffering*.

The participants’ ability to cope was significantly influenced by the staff’s intervention. The nurses’ additional endeavours to care for the patients in the absence of their relatives, such as organizing window visits or phone calls, were highly valued. Consequently, the relatives experienced emotional empathy and saw open and transparent communication as beneficial.

P8: *The professionals were very attentive and open to the possibility of video calls, even when he* (grandfather) *was less reactive, when he heard the voice of someone familiar, he reacted. There were even times when they allowed contact through the ward window. They were very important moments*.

P2: *Video calls allowed us to be with the rest of the family… because we felt that she* (mother) *felt everyone’s presence, because she listened to them. And for my brothers it was also good, because although they couldn’t go there, they could see it. And what the eyes see, the heart feels.*

P9: *It’s funny that I got in touch with a nurse who was sending me some photos of her* (wife) *sewing, I don’t know why, to occupy her time… they were fantastic.*

Some individuals expressed gratitude toward professionals allowing significant actions, such as bidding farewell to a loved one, making physical contact, maintaining eye contact, or caressing their hair. Likewise, they were attentive to family needs.

P6 asserted: *For me, the people who work in palliative care, it doesn’t mean that they aren’t the same on other sides, they are angels without wings, but they fly… He* (husband) *was treated with professionalism, with humanity, with affection, with everything he deserved.*

P11 also added: *At no point did I feel like I was a team and we… no, we were one. When I was visiting, they allowed me to participate in the care, which was very important to me. Furthermore, they were worried about whether I rested, ate, or whether I went outside to get some air.*

In contrast, two participants were sceptical about which care was system-led, which was not aligned with the holistic paramount of palliative care. Their “fight” appeared to be a means of reestablishing a sense of control and seeking purpose, such as restoring the dignity of their loved one or resisting feelings of powerlessness in the presence of suffering.

P5: *I felt some lack of integration between health, social and spiritual care. I needed that my relative have the presence of a priest and they told me that was not possible.*

P13: *I remember my husband had recurring muscle pain and they gave him morphine. When I asked if it was possible for the physiotherapist to do some exercises for his comfort, they said no because he had other priorities at the time. I soon thought how the physiotherapist could have other priorities if he hadn’t evaluated him yet. At these moments a person feels helpless.*

Two other participants were ambiguous about the caring circumstances of their loved ones. P3 noted the following: *The nurse told me that if we wanted to see my father alive, we would have to expedite the visit as quickly as possible. And that’s when we had to carry out tests for SARS-CoV-2, but the pharmacies didn’t have any vacancies… it was a race against time. In the end we didn’t arrive on time, it would have been better if they hadn’t told us anything.* In parallel, P1 found similar constraints to obtain the tests for SARS-CoV-2: *I even got angry with the doctor, after all my husband was dying and I wanted to see him for the last time.*

#### 3.2.2. The Last Farewell Was Robbed

Participants’ experiences before the death of their loved one varied slightly based on the feasibility of bidding farewell. A subset of the participants personally witnessed the moment of their loved one’s passing. Some individuals were permitted to bid farewell, while others were deprived of the chance to say their goodbyes altogether. Emotions of grief, anger, and powerlessness were experienced. The participants’ ability to cope with the loss of their loved one was significantly influenced by the social and family networks. Those who perceived support during this vulnerable time found it easier to relate to their situation, while participants who felt uncared for experienced greater strain.

(a) Missed opportunities to be together

The absence of physical contact and emotional proximity with loved ones made their grieving experience appear unnatural. This made it more challenging for them to share memories, freely discuss their feelings, and start the process of accepting and dealing with their collective grief and loss. Additionally, it was more challenging to obtain or offer the necessary emotional assistance, resulting in relatives experiencing feelings of isolation and disinterest in daily life activities.

P14: *We were allowed to be present in the last hours of their lives, I will never forget that moment. I had the chance to say goodbye. But my brother didn’t have that possibility, and I realize how much he suffers, he isolated himself, I have to support him but it’s hard.*

P5: *I stopped going out because I don’t feel comfortable going out alone. Where am I going alone? My mother already has difficulty with mobility too, she is always in bed. The pain remains and I ended up losing the habits that were good for me*.

P1 adds that he stopped doing some activities due to loss of interest: *I feel numb since my husband’s death. In the past, I wouldn’t go to bed on Saturday without my house being completely clean. Now, look, everything going on here, everything is like this, once I clean one thing, again I clean another…*

Individuals also expressed concerns about the additional anguish resulting from the necessity of independently commuting to and from funerals, being seated separately from loved ones, and the inability to provide or receive solace from them.

P12: *The funeral was just for the family, but two or three of my friends ended up attending. But with the risk of contamination, we couldn’t even touch each other, there were no hugs and how much I missed them.*

Relatives struggled with a sense of meaninglessness and lack of purpose. This pertained not only to their estranged relationship and the corresponding duties, activities, and life plans, but also to the challenges imposed by the disruption of familiar routines and relations.

P12: *I lived for the sake of living, I had no love for life, I stopped working, I had no life goals… I had nothing… let it go, it was just another day. At night, I often cried, cried, I felt like breaking everything.*

P15: *Sometimes I feel like giving up. On the day of the funeral, everyone went to hug the family, and I was there in the corner, alone… only my daughter came to hug me. But first she hugged her godmother and the whole family, but then she saw that I was alone there with my husband, in the corner, and she came to me.*

P14: *That’s how I lived it all alone at home. No one from the family called me to ask if I needed anything.*

P16 perceived that she was subjected to negative evaluations because of her sub-par performance, resulting in feelings of inadequacy, as outlined in the following statement: *I had anxiety attacks, to the point of having difficulty working. I’ve already thought about changing jobs, I don’t have the patience to put up with others, as my performance has decreased, and my boss isn’t worried in the slightest.*

There were noticeable alterations in the way people collectively grieve, characterized by *a diminished sense of communal solidarity and togetherness*, as stated by P10. Some communities united in solidarity to support families in need. Various local shows of support were described, one of which involved a hearse transporting a deceased patient’s body to the burial site.

P3: *We contacted one of the neighbours to join us, although all of them expressed their desire to come, they were unable to do so. We informed them that we intended to visit the house one final time before proceeding to the cemetery. Subsequently, they congregated on the front lawn to bid farewell.*

(b) Restrictions in making legacy and memorialization

Grieving families consider the preservation of their relative’s legacy to be a crucial part of their coping mechanism and the process of finding meaning in their loss. Indeed, two participants stated that their ill family members intended to be remembered (willing away belongings, writing letters to loved ones), but pandemic restrictions made accomplishing their desires and wishes impossible. P7 said, *we were unable to clothe her, style her hair, or place small items in the coffin or conduct a proper wake for her*.

For P15, what disturbed her most was when the undertaker told her the deceased bodies were not dressed, stating the following: *they were just placed inside two black bags. My brother and I didn’t want to believe it would be like this. What an infamous act this was…*

The bereaved family members were further distressed by the limited funeral traditions, particularly when just 10 or fewer individuals were allowed to attend, and the customary activities like readings and singing in the catholic tradition were forbidden.

P2: *Only ten people were allowed, there was no singing, and even ringing the bell wasn’t allowed, because they called people. However, I spoke to the priest, because for my father it was sad not to even hear the bell, because it was the announcement to the community that someone had passed away. The priest allowed the bell to ring at the end of the celebration.*

Disruptive memorialization practices persisted after the funeral. The imposition of limitations on visiting cemeteries resulted in an additional source of anguish. P14 also expressed how the pragmatic and managerial aspects of coping with their loss had become more challenging due to the interruption caused by the pandemic to professional services. These tasks encompassed *acquiring the death certificate, coordinating funeral remotely and notifying financial institutions of the deceased’s passing*.

Despite these constraints, some family members openly recounted their recollections of the characteristics of the dead relative. P2 discussed the attributes of his deceased mother in the following statement: *The image I keep of my mother is the one from when she went to Palliative Care, where she smiles, where she still talks. That’s the image I keep.* P3 also stated the following: *My father left us many teachings and many legacies that we also try to pass on to our children and that we kept. But everything was also resolved… it was a cycle that was closed.*

#### 3.2.3. Looking for Adjustment after Loss

The COVID-19 pandemic altered the grieving process. The physical absence was prevalent, and the grieving family was often physically separated from the deceased’s corpse. Occasionally, family members experienced anger over being unable to be present with the dying individual and their remains following their passing during the pandemic. This can lead to a sense of disconnection from the world and a desire for concrete proof of the loss. However, the search for a return to life is one of the main challenges for the bereaved.

(a) Oscillating between good days and bad days

The incapacity to visit or bid farewell caused a profound sense of sadness and regret in several participants, as they were unable to be present to console and assist their loved one. In bad days, relatives recall their inability to be physically present with the loved one at their time of death. Not being present at the time of death was found to be a highly distressing responsibility for bereaved family members and had a negative impact on their overall state of wellbeing.

P1: *I wasn’t there when he died, he needed me. On the worst days… in bed, I think I’m alone… I no longer sleep even with the pill… I no longer sleep.*

P5: *I avoided going to the cemetery for a while, because I’m sick. After all, I wasn’t with him when he needed it most (death)… it’s the nostalgia, it really affects me… I miss it a lot, he was a true companion.*

P13 expressed a feeling of having let others down and was concerned that others may have felt neglected: *I didn’t go to bed for two months; it was the bed where I slept with him. I slept on the couch because I had nightmares, with the feeling that he wasn’t okay because I defrauded him when he needed me most.* P16 also added that she continues to wait for him to return because she did not have the opportunity to confirm that he had passed away, which creates a permanent disconnection with reality: *I just saw a sealed coffin… Could it be him? That’s why I didn’t take anything from the place, it gives me the feeling that he might come back.* Similarly, P15 underlined her need to have a concrete proof of death: *We heard a lot of things, the exchange of bodies, of so-and-so swapping with so-and-so… I don’t think about it… but there is this doubt, in his place, there could be someone else.*

In addition, P1 says that home has come to represent a space where he does not feel good, stating the following: *It’s hard for me to be in this house, it’s become an empty nest. I don’t feel cozy, I feel disconnected, alone.* Other participants, like P12, stated that commemorative dates (e.g., Father’s Day, Christmas) have lost meaning for the bereaved relatives because the deceased is no longer present: *Christmas doesn’t mean anything to me, it’s a day like any other, there’s someone missing at the table, and they will always be missing… it doesn’t mean anything. But Father’s Day hurts me even more, it will always hurt…*

On good days, bereaved relatives discuss the ways they maintain a continuing bond with their loved one, either through a sentimental object or by honouring their legacy. P4 stated the following: *I feel a sense of loss when not wearing a charm, as it symbolizes the presence of their loved one in their heart.* From this perspective, affirmations of life were strongly associated with a wish for others to remember their deceased relatives: P8 stated the following: *On certain occasions, I am grateful for possessing these mementos as they serve as a reminder of her existence and ensure that I am mindful of her presence*. P13 recalled the suffering her husband was in, and death represented relief, which helped to deal better with the loss: *He was in great suffering. He was suffering a lot. We don’t want them to leave, but it was better this way.*

(b) Accepting harsh truth

The participants’ ability to cope with grief was affected by the loss of their usual routines and structure. They tried to return to their normal lifestyles, revealing the possibility of finding positive aspects in the altered grieving process. Several participants said that they were able to *reinterpret their experience of loss* (P8). They experienced a *sense of tranquillity* (P4) and found *an opportunity to reflect and make sense of things* (P8). These steps provided protection from others, allowing them to establish and uphold their own boundaries. This ensured that their experience remained unaffected by the experiences of others in their family.

Participants expressed pleasant experiences in the development of novel rituals, where they discovered *resilience and assistance in contemplating innovative possibilities and methods* (P8). The process of creating new rituals was characterized as a profound encounter, enabling participants to discover more symbolic methods of uniting and imparting a sense of purpose. This experience proved especially valuable during a period when they felt powerless.

Bereaved relatives resorted to psychological support if needed. The palliative care team’s support, through letters of condolence or phone calls after the death of a loved one, was highlighted as positive measures to mitigate the post-loss period. P15 stated the following: *I didn’t even know that there was this support to help… it was the phone calls and a letter that arrived… then I was offered psychological support. After all, it’s not just death, it’s other things that come after… my brothers, my family, and I feel alone. At the psychology consultation, I have space to be heard and this is very important for my mental health.* P12 also stated the following: *When they sent me a letter to find out if we wanted psychological help, I came right away, I noticed that I wasn’t feeling well. I lived for the sake of living, I had no love for life, I had no goals, I had nothing…*

Lastly, some participants referred to the inexorability of death as an integral part of life and how this helped them accept death, even if sometimes with resignation. In this regard, P3 states the following: *Even my father prepared us for this, he said “Look, don’t cry, … because this is normal. Then it will happen to you too and you… have to accept it, there is no other way solution if they don’t accept it.”* P9 also stated the following: *We talked openly about death and that it would be close, but I think there is always something left to say and do. However, this will always be part of our lives, regardless of these situations.*

## 4. Discussion

This work is one of the first phenomenological studies of the experiences of bereaved relatives of patients who died in PCUs during the COVID-19 pandemic in Portugal. This study generated an in-depth understanding of the challenges faced by bereaved families of patients in PCUs under COVID-19 pandemic regulations.

One of the findings of this study pertains to the pre-death conditions of the palliative patients. In line with previous evidence, the narratives underlined not being by the patient’s side, failure to perform religious rituals at the patient’s bedside, anxiety around the news of death, adherence to public health restrictions, and the prohibition of viewing the deceased’s body [45,57]. The disruptive trajectory of family relatives induced significant distress and rendered them unable of acknowledging the patient’s death and coping with loss [58]. This may have delayed the process of bereavement, leading to suspended or delayed grief [57,59]. Evidence also suggests that prohibition of mourning rituals can predispose people to complicated grief syndrome or persistent complex bereavement disorder [60], especially among those bereaved after an unexpected death (like COVID-19) and those who had a close relationship with the deceased [61].

Following the COVID-19 pandemic, swift measures were implemented to address the ambiguity surrounding the disease. These procedures had the unintended consequence of limiting communication between patients/families and healthcare personnel, and also hindered the ability to carry out planned actions. As a result, the idea of being prepared for death underwent a significant change [62]. COVID-19 presented novel obstacles that impacted the readiness for dying in terms of emotional, physical, and spiritual resources. The lack of preparedness for death arises as a transitional phase and a complex process marked by ambiguity and unpredictability over the course of the disease. This leads to feelings of anxiety and an inability to effectively address existential concerns in order to ensure a peaceful and good death [13].

As previously documented in other studies, our findings emphasize the significance of relatives’ requirement for information and efficient communication from HCPs [16,36]. Overall, participants enjoyed the information provided by HCPs; however, they sometimes found it scarce. Mulcahy et al. [63] suggest that a knowledgeable family member can contribute to the decision-making process in PCUs when the patient is unable to communicate, effectively serving as an extension of the patient. According to Wang et al. [64], effective communication was crucial in delivering EoL care amidst the COVID-19 pandemic. In addition, good communication with healthcare staff promotes a sense of control influencing preparedness for the caregiving journey [65].

The pandemic made it clear that physical proximity was no longer guaranteed, highlighting the significance of being physically present during someone’s final moments and the impact it had on the experience of bereavement when that choice was not available. Relatives reported regret and a sense of forsaking the dying individual, as their loved ones passed away in isolation [66,67]. The inability to be physically there at the time of death had a significant impact on the interpretation of the deceased person’s death by grieving family members. This element played a crucial role in the stress experienced by relatives during the pandemic [68]. The individuals perceived their farewell as inadequate, due to the prohibition of physical contact or proximity with their beloved [69]. In certain instances, this resulted in refusal to acknowledge the loss [66].

Providing companionship to someone in their final moments was determined to be crucial for family members, as it facilitated their process of reaching emotional resolution, particularly if they were able to visit [67,68,69,70]. Despite the limitations imposed by measures such as social separation, PPE kit, and the inability to physically interact, the existence of social support, even if not in a physical form, was found to be significant. Families expressed gratitude for the increased visiting options during EoL [37]. However, some families believed that these possibilities were provided too late, as their loved ones were no longer responsive at that time [68]. Our findings also found that adapted goodbye rituals via videocalls were valorised by participants, despite ambivalence regarding their sufficiency. In this vein, mixed emotions, in which people simultaneously experience positive and negative emotions, dominated the emotional landscape [71] of many individuals during the bereavement trajectory. Such ambivalence reflects some inconsistencies in care where bereaved families have expressed appreciation for the sacrifice and efforts of HCPs as well as perceived a low level of interaction quality, communication, and perceived compassionate care [72].

Closeness throughout the final stages of life offers solace and fosters emotional connection. The findings of our study demonstrate the profound sense of powerlessness and emotional detachment experienced by family members who were granted visitation rights but were prohibited from engaging in physical contact with the patient. The relatives emphasized their desire for a harmonious balance between safeguarding against infection and a respectful farewell [73]. Within our research, this balance is significant in the context of bidding farewell during the pandemic, particularly in light of the absence of physical contact during EoL as a result of concerns regarding infection.

Our study also shows that families valued other forms of assistance, such as psychological support after loss. Some relatives went to psychological consultations as a strategy to better manage their family member’s death and the surrounding circumstances. Hence, it is imperative to provide access to grief counselling and support groups in presential or virtual ways for family members and aggressively advocate for their utilization [74,75]. If a visit to a patient is not feasible, PCUs should provide an opportunity for discussions with the healthcare professional. These discussions should focus on the patient’s EoL, as well as their medical and psychological state, to perhaps mitigate the unsettling nature of dying. Bereaved relatives require both private and public moments to communicate their grief in order to determine the significance of the deceased’s life and death [23].

The available evidence indicates that the shifts in both the location of death and the recipients of palliative care were not directly caused by the COVID-19 infection itself [76,77]. Rather, these changes were a result of indirect effects of the pandemic, such as alterations in service accessibility and disruptions to the healthcare system. Consequently, researchers suggest allocating resources towards primary, community, and palliative care services in order to guarantee exceptional and fair EoL care in response to increasing demands [77]. Nevertheless, our research indicates that the pandemic crisis necessitated a new and alternative method of delivering EoL care, emphasizing fragmented care rather than the comprehensive approach typically associated with high-quality palliative care. In this sense, it is essential to reinforce the words of Dame Cicely Saunders that families, like patients, matter [78]. In the realm of human suffering, families appreciate a non-abandonment attitude that creates a sense of belonging and a feeling of being valued. Likewise, a shared sense of meaning and purpose gives a tone of care where dignity is valued. Lastly, therapeutic humility offers the surest path toward healing, which requires an ability to relinquish the need to fix, offering an optimal response to their loved one’s anguish and providing comfort [78].

### 4.1. Strengths and Limitations

One strength of this study was to extend the available knowledge from qualitative data material gathered from 16 bereaved family members. Another strength was the incorporation of several detailed descriptions in the form of quotations (detailed accounts of meanings). The inclusion of these verbatim quotations enhanced the transparency and trustworthiness of the interpretation by revealing the researcher’s thought process during data analysis and how it impacts the development of codes. Having a registered nurse and psychologist who worked in palliative care for several years on the research team was important to critically discuss the findings. Two additional members of our research team (despite their limited experience in palliative care units) brought extensive expertise from analogous environments, as well as collaboration in data analysis, coding and critical thinking. This mix of competencies and perspectives helped the team avoid unidimensional interpretation of findings, thereby improving trustworthiness through researcher triangulation.

Notwithstanding the merits of this study, there were certain drawbacks. Initially, the participants’ accounts primarily revolved around occurrences from November 2021 to June 2022, a period characterized by reduced public health interventions and limitations, in contrast to the early stages of the pandemic. These circumstances could potentially influence our research findings. Although the study is limited to the experience of relatives of people who died mainly in the central region of Portugal, our findings offer transferable insights to other similar contexts. Findings were restricted to relatives whose family members passed away in a hospital and do not reflect the experiences and requirements of families when a family member died in other environments, such as a home or nursing home, despite efforts to include these relations. Whereas substantial effort was expended in developing a credible sample, the study secured a small purposive sample, which increases the likelihood that certain experiences were not captured by the study. Furthermore, the sample consisted predominantly of females, which accurately reflects the fact that most family caregivers are women [79]. Additionally, we only focused on grief in adults in a single period; other studies could include narratives from bereaved children and adolescents from a longitudinal perspective. Using large and diverse samples, future studies might employ a mixed methods approach to investigate the connections between the presence of categories of meaning-making derived from the qualitative data and the grief outcomes. Further work should also include perspectives of grief other than those of bereaved relatives, such as healthcare professionals or other members of society. While participants’ memories of the deaths of their loved ones are still fresh, some recall bias impacted the free flow of ideas. Moreover, our findings should be limited to the context of Portuguese culture, given that it is widely acknowledged that grief can be influenced by cultural factors. It is advisable to conduct research on how COVID-19 affects grieving in different cultures and contexts.

### 4.2. Implications for Practice

The participants stressed the significance of assistance, information, and follow-up. Thus, HCPs must recognize the significance of compassionate communication and consistent assistance, even in the face of challenging circumstances. Nevertheless, additional investigation is necessary in this domain with respect to analogous circumstances. Furthermore, it is crucial that family members can visit their loved ones at the hospital to the greatest extent feasible, and actively participate in their treatment and decision-making processes. Emerging ethical issues concerning the EoL decisions of loved ones have highlighted the need for future studies to focus on healthcare professionals prioritizing communication with patients and their relatives.

To effectively prepare for future pandemics, it is crucial to prioritize creating opportunities for personal contact with those who are terminally ill and nearing the end of their lives, or to provide proactive alternatives that ensure dignified EoL care. Engaging in activities that provide a lasting legacy can help families, particularly children, maintain a connection with their deceased loved one. Examples of such activities include preserving handprints or creating hand moulds of the patient, as well as obtaining a cardiac tracing from the patient’s final days to send to the family [75]. Other strategies to facilitate post-loss adjustment include the following [26,80]: (a) offering psychoeducational resources on the topic of grief; (b) promoting the establishment of a regular schedule, prioritizing self-care activities such as medical check-ups, and utilizing technology to maintain social connections; (c) assisting in the restructuring of ruminative thought patterns; and (d) referring individuals to professional grief counselling services.

We also recommend that policymakers allocate additional resources to palliative care services to ensure the provision of exceptional EoL care for patients and their families, with the aim of better preparing for future pandemic events. Our findings contribute to the development of national bereavement response strategies in the aftermath of the pandemic. Stepped national bereavement response strategies may be beneficial in preventing complications, as the pandemic affected all clients who experienced grief and bereavement during this period. National meaning-making practices are advised as an initial measure because they allow the progression of a grief trajectory [81]. These may enable people to participate in the ritual and collective lament that was unavailable due to pandemic public health measures, thereby allowing them to commemorate the lives of those who have passed away during the pandemic. Furthermore, policy changes may entail the implementation of training programs for healthcare personnel, enhancement of communication strategies, and promotion of advanced care planning efforts.

## 5. Conclusions

Our findings illustrate the remarkably challenging experiences of bereavement during the pandemic, which are characterized by a substantial disruption to social support networks, as well as EoL, death, and bereavement practices. The participants’ lived experiences were summarized in the following three categories: (1) navigating the loved ones’ final weeks and days (troubled deaths); (2) the last farewell was robbed; (3) looking for adjustment after loss. From these categories, one overall main theme emerged, which was as follows: “Struggling between stolen moments and painful losses to get back into the flow of life”. Our findings suggest that developing and promoting family-centred culture can lead to compassionate palliative care focused on a myriad of ways of affirming that their loved one matters.

## Figures and Tables

**Table 1:** Participants’ background (N = 16).

Participants	Age	Sex	Education Level	Type of Bond with the Deceased	Family Support	Significant Previous Losses
**P1**	60	Female	9th grade (3rd cycle of basic education)	Spouse	Yes	Yes
**P2**	43	Female	12° grade (secondary education)	Daughter	Yes	Yes
**P3**	43	Female	12° grade (secondary education)	Daughter	Yes	Yes
**P4**	57	Female	12° grade (secondary education)	Daughter	Yes	Yes
**P5**	57	Female	12° grade (secondary education)	Spouse	Yes	Yes
**P6**	72	Female	4th grade (1st cycle of basic)	Spouse	Yes	Yes
**P7**	56	Female	Licentiate’s Degree	Daughter	Yes	No
**P8**	28	Female	Licentiate’s Degree	Grandchild	Yes	No
**P9**	52	Male	Licentiate’s Degree	Spouse	Yes	Yes
**P10**	36	Male	Licentiate’s Degree	Boyfriend	Yes	No
**P11**	32	Female	Licentiate’s Degree	Girlfriend	Yes	Yes
**P12**	22	Male	9th year (3rd cycle of basic education)	Son	Yes	Yes
**P13**	55	Female	4th year (1st cycle of basic education)	Spouse	Yes	Yes
**P14**	49	Female	12° grade (secondary education)	Daughter	No	Yes
**P15**	47	Female	6th year (2nd cycle of basic education)	Daughter	No	Yes
**P16**	47	Female	Licentiate’s Degree	Daughter	Yes	No

## Data Availability

All data generated or analyzed during this study are included in this article. This article is based on the first author’s master’s dissertation in Palliative Care at the School of Health Sciences—Polytechnic University of Leiria.

## Supplementary Materials

The following supporting information can be downloaded at https://www.mdpi.com/article/10.3390/healthcare12171763/s1, Supplementary Table S1: Consolidated criteria for reporting qualitative studies (COREQ): 32-item checklist.
