# Supplementary material for: Family Experiences of Loss and Bereavement in Palliative Care Units during the COVID-19 Pandemic: An Interpretative Phenomenological Study
Source: Healthcare (Basel). 2024 Sep 4;12(17):1763. doi: 10.3390/healthcare12171763 (PMC11395245; doi:10.3390/healthcare12171763)
Supplement: Supplementary file 1 [file healthcare-12-01763-s001.zip › healthcare-3179371-supplementary.pdf]

Supplementary Table S1 - Consolidated criteria for reporting qualitative studies (COREQ): 32-item checklist

No.	Item	Guide questions/ description	Checklist of this study
**Domain 1: Research team and reflexivity**			
**Personal Characteristics**			
1.	Interviewer/facilitator	Which author/s conducted the interview or focus group?	One author (M.J.M.).
2.	Credentials	What were the researcher's credentials? E.g. PhD, MD	BSc
3.	Occupation	What was their occupation at the time of the study?	Interviewers, data analysts, writers and revisers of first drafts of the paper.
4.	Gender	Was the researcher male or female?	Female
5.	Experience and training	What experience or training did the researcher have?	The interviewer received training in qualitative research methods as part of an ongoing Master's project.
**Relationship with participants**			
6.	Relationship established	Was a relationship established prior to study commencement?	No
7.	Participant knowledge of the interviewer	What did the participants know about the researcher? e.g. personal goals, reasons for doing the research	Reasons for doing the research.
8.	Interviewer characteristics	What characteristics were reported about the interviewer/facilitator? e.g. Bias, assumptions, reasons and interests in the research topic	Reasons and interests in the research topic.
**Domain 2: study design**			
**Theoretical framework**			
9.	Methodological orientation and Theory	What methodological orientation was stated to underpin the study? e.g. grounded theory, discourse analysis, ethnography, phenomenology, content analysis	Phenomenology
**Participant selection**			
10.	Sampling	How were participants selected? e.g. purposive, convenience, consecutive, snowball	Purposive and snowball sampling techniques

11.	Method of approach	How were participants approached? e.g. face-to-face, telephone, mail, email	Face-to-face
12.	Sample size	How many participants were in the study?	16
13.	Non-participation	How many people refused to participate or dropped out? Reasons?	No
**Setting**			
14.	Setting of data collection	Where was the data collected? e.g. home, clinic, workplace	Two Palliative Care Inpatients Units (central region of Portugal)
15.	Presence of non-participants	Was anyone else present besides the participants and researchers?	No
16.	Description of sample	What are the important characteristics of the sample? e.g. demographic data, date	Demographic data
**Data collection**			
17.	Interview guide	Were questions, prompts, guides provided by the authors? Was it pilot tested?	Yes
18.	Repeat interviews	Were repeat interviews carried out? If yes, how many?	No
19.	Audio/visual recording	Did the research use audio or visual recording to collect the data?	Yes
20.	Field notes	Were field notes made during and/or after the interview or focus group?	Yes
21.	Duration	What was the duration of the interviews or focus group?	Average of 72 minutes (ranging from 30 to 145 minutes)
22.	Data saturation	Was data saturation discussed?	Yes
23.	Transcripts returned	Were transcripts returned to participants for comment and/or correction?	Yes
**Domain 3: analysis and findings**			
**Data analysis**			
24.	Number of data coders	How many data coders coded the data?	Two

25.	Description of the coding tree	Did authors provide a description of the coding tree?	Yes
26.	Derivation of themes	Were themes identified in advance or derived from the data?	Derived from the data
27.	Software	What software, if applicable, was used to manage the data?	WebQDA software
28.	Participant checking	Did participants provide feedback on the findings?	No
**Reporting**			
29.	Quotations presented	Were participant quotations presented to illustrate the themes / findings? Was each quotation identified? e.g. participant number	Yes
30.	Data and findings consistent	Was there consistency between the data presented and the findings?	Yes
31.	Clarity of major themes	Were major themes clearly presented in the findings?	Yes
32.	Clarity of minor themes	Is there a description of diverse cases or discussion of minor themes?	Yes

Tong, A.; Sainsbury, P.; Craig, J. Consolidated criteria for reporting qualitative research (COREQ): A 32-item checklist for interviews and focus groups. *Int. J. Qual. Health Care* **2007**, *19*, 349-357. https://doi.org/10.1093/intqhc/mzm042
